# Health workforce response to Covid‐19: What pandemic preparedness planning and action at the federal and state levels in Germany? Germany's health workforce responses to Covid‐19

**DOI:** 10.1002/hpm.3146

**Published:** 2021-03-18

**Authors:** Julia Köppen, Kimberly Hartl, Claudia B. Maier

**Affiliations:** ^1^ Department of Health Care Management Technische Universität Berlin Berlin Germany; ^2^ Medical Department Division of Gastroenterology and Hepatology Charité – Universitätsmedizin Berlin Berlin Germany; ^3^ Max Delbrück Center for Molecular Medicine Berlin Institute for Medical Systems Biology Berlin Germany

**Keywords:** Covid‐19, decentralised governance, Germany, health workforce, skill‐mix, surge capacity

## Abstract

**Introduction:**

The Covid‐19 pandemic has required countries to prepare their health workforce for a rapid increase of patients. This research aims to analyse the planning and health workforce policies in Germany, a country with a largely decentralised workforce governance mechanism.

**Methods:**

Systematic search between 18 and 31 May 2020 at federal and 16 states on health workforce action and planning (websites of ministries of health, public health authorities), including pandemic preparedness plans and policies. The search followed World Health Organisation (WHO) Europe's health workforce guidance on Covid‐19. Content analysis was performed, informed by the themes of WHO.

**Results:**

The pandemic preparedness plans consisted of no or limited information on how to expand and prepare the health workforce during pandemics. The 16 states varied considerably regarding implementing strategies to expand health workforce capacities. Only one state adopted a policy on task‐shifting despite a federal law on task‐shifting during pandemics.

**Conclusions:**

Planning on the health workforce, its capacity and skill‐mix during pandemics was limited in the pandemic response plans. Actions during the peak of the pandemic varied considerably across states, were implemented ad hoc and with limited planning. Future action should focus on integrated planning and evaluation of workforce policies.

## 1 INTRODUCTION

The Covid‐19 pandemic has put many health systems under pressure across the world. A first wave hit most countries in the spring of 2020, followed by a second wave in the winter of 2020/2021. Although nearly all countries worldwide were and are affected, the extent varied considerably. As of 11 November 2020, the highest number of total infections existed in the following six countries: the United States (US), India, Brazil, France, Russia and Spain.^1^, ^2^ Other countries, such as Italy, showed high numbers of severe cases in short periods of time in regional hotspots, such as in Lombardy region.^3^, ^4^ Country responses to the Covid‐19 pandemic have been shown to vary, but often included several measures to avoid transmission and to increase health system capacity. Health system capacity is closely linked to a country's health workforce capacity and sustainability.^5^, ^6^, ^7^ According to the World Health Organisation (WHO), a well‐trained health workforce in sufficient numbers with the possibility for expanding capacity is critical for an effective response to Covid‐19, among other health system‐related factors.^8^

Many countries experienced acute shortages of health professionals to treat the high number of patients with Covid‐19. In the US, several hospitals in New York City reacted to staff shortages and expanded their workforce. Measures included redeployment and training of existing staff, hiring private staffing agencies and making use of volunteers.^7^ In Italy, a shortage of health professionals was reported as a result of years of austerity measures and staff, for example, doctors migrating to other countries. This situation exacerbated, for instance in the Lombardy region, when high numbers of patients with Covid‐19 had to be treated.^3^ As a response, rapid actions were undertaken, such as redeployment of staff, hiring of foreign‐trained doctors, early graduation of students, among others (ibid). Some measures were considered to ease restrictive regulations in the US to allow new skills‐mixes and task‐shifting, but progress was overall small.^9^

The WHO Regional Office for Europe has provided recommendations for countries to strengthen health systems, including training, repurposing and mobilising sufficient health professionals during the Covid‐19 pandemic.^8^ Measures include identification of free health workforce available for surge capacity, skill‐mix and repurposing of health professionals for rapid deployment, addressing contractual changes such as adjustment of working hours (e.g., part to full time) and scope of work, maintaining ongoing communication with health professionals, and protecting the physical health and safety of the frontline workers.^8^ A cross‐country comparison of 36 countries in Europe and Canada found several workforce‐related policy measures taken to support the health workforce and overcome staff shortages.^5^ Examples include additional financial compensation or bonuses, mental health support, as well as practical support such as childcare or free transport (ibid). The authors found that overall, evaluations were lacking to determine the effectiveness of the interventions. Another study across 44 countries in Europe and Canada described measures to expand the health workforce. Common strategies were to augment the capacity of available health professionals and the recruitment of medical and nursing students.^10^ An OECD report identified large cross‐country variations in the policy responses to the epidemic.^11^ Countries that were found to react effectively during the spring of 2020 were Finland, Norway and Estonia from within Europe. Suggested reasons were geographical features such as lower population density, the rapid implementation of containment strategies as well as trust and adherence to these measures by citizens. In Finland, shortages of medical materials and supplies were prevented because of sufficient stockpiling (ibid).

In Germany, the first Covid‐19 case was confirmed on 27 January 2020.^12^ Within the following four months until the end of May, measures were planned and implemented at federal and regional level. During this time the Federal Ministry of Health suggested hospitals to recruit more staff and to cancel elective procedures to increase the capacities to take care of patients with Covid‐19.^13^ Health professionals in hospitals had to adapt quickly to the new circumstances. A qualitative study based on views of nursing managers and nurses specialized in hospital hygiene showed that skill‐mix had changed and additional training was needed (among others).^14^ Health professionals are at high risk for being infected; as of May 2020, an over proportional number of all infected persons worked in hospitals, medical practices, dialysis facilities and emergency services (7%).^15^

The Global Health Security (GHS) index of 195 countries analysed country responses to epidemics. It showed large differences in countries' capacities to react to infectious disease outbreaks.^16^ Germany ranked 14th overall globally, based on six indicators: prevention, early protection, rapid response, health system capacity, national policy capacity and risk environment. Yet, Germany ranked only 22nd in terms of health system response. This ranking is based on countries' overall capacity to react to biological threats, including infectious disease outbreaks, and was not specific to Covid‐19.

There is a paucity of research on countries' health workforce responses to Covid‐19.^17^ The two mentioned cross‐country comparisons and the OECD report covered health workforce strategies from an international perspective drawing early lessons,^5^, ^10^, ^11^ but lacked in‐depth analyses of individual country responses. All three studies looked at primarily federal‐level responses to the pandemic. Yet, many countries, such as Belgium, Canada, Germany, Italy and the US, have a devolved, decentralised governance of their health systems, including during times of pandemics and health crises. There is limited research analysing the responses of countries with decentralised governance systems. According to Greer et al.^18^ an in‐depth analysis of policy, politics and governance systems of individual countries is required to understand countries' responses to the pandemic.

The aim of this article was to analyse Germany's response as a large, highly populated country with a decentralised health system governance during the first wave of the Covid‐19 pandemic, taking account of policies and actions planned and implemented at the federal level and by the states. Germany consists of 16 states with a ministry of health each, plus a Federal Ministry of Health.^19^ Key actors include the public health authorities which are available at the federal, state and municipality level. The responsibilities at state level concern public health, such as the prevention and control of communicable diseases, including during pandemics, and other health‐related decisions, for instance on inpatient care.^20^ The Federal Ministry of Health provides overall guidance, for instance, by means of laws (e.g., the infection control law) and directives, which are then adopted by the states. The Robert‐Koch‐Institute (RKI) serves as the government's central scientific institution on public health and pandemic responses. Hence, Germany is an example of a country with a devolved, multilevel governance system.

## 2 METHODS

The study is based on a systematic search at federal and regional levels of all 16 states on health workforce action and planning regarding the Covid‐19 pandemic during the first wave which took place in the spring of 2020, following WHO's guidance on health workforce responses.^8^

This article focusses on two thematic areas derived from the WHO's guidance: (i) increase in workforce capacity and (ii) skill‐mix and task‐shifting. The two thematic areas were chosen, because they comprise key elements to strengthen surge capacity and flexibility of the health workforce during epidemics. According to the WHO, increase in workforce capacity includes temporary measures such as expanding working hours, reallocation of staff from nonaffected areas, employment of retirees, medical and nursing students, military and nonpracticing healthcare workers (ibid). Skill‐mix and task‐shifting are defined as changing roles, tasks and competencies of health professionals (skill‐mix) and shifting tasks and roles from a higher to a lower qualified health professional (task‐shifting). The two concepts encompass among others rapid training (up‐skilling) of workers, expanding scope of practice for health care workers, implementation of supervision structures, but also expedite training and early certification for medical and nursing students (ibid). The aim was to identify if and to what extent Germany implemented the strategies at federal and state level.

The websites of the federal and state ministries of health and public health authorities were systematically accessed and searched for any of the two above‐mentioned thematic areas. To this end, the websites were screened, and all written documentation systematically analysed, including (i) the pandemic preparedness plans and (ii) policies and other measures specific to the health workforce. First, the pandemic preparedness plans were included in the analysis to assess if and to which extent the health workforce was taken into account in the planning phase prior to the pandemic. Second, the search also encompassed legal documents (laws, bylaws, decrees) and other policies mentioned on the federal or regional levels if directly related to the health workforce and Covid‐19. Inclusion criteria were information provided in written form directly on the websites or via hyperlinks. Excluded were information provided in video, audio or exclusively on social media. Snowballing was applied in addition to the systematic search, which involved following hyperlinks to other websites or documentation if assessed as directly related to the topics of interest. The search was performed by three researchers after a pilot phase, which was applied to ensure that all researchers followed the same procedure. During the pilot phase a selection of three states was chosen to test the search strategy, discuss uncertainties and seek consensus.

The final search was carried out between 18 and 31 May 2020, which was towards the end of the first wave of the pandemic. The timing was deemed appropriate, as most Covid‐19 related workforce policies and measures taken during the first wave were available on the websites by then. Each researcher conducted the search for five or six states, respectively, and extracted the findings in a condensed form including references in an Excel template which was based on the WHO's topics. The search on federal level policies and measures and pandemic preparedness plans was conducted by one researcher. As the content of websites can change over time, all sources were saved during the search.

Content analysis was performed, informed by the themes of WHO, using a deductive approach (themes and subthemes are defined a priori). The underlying contents were extracted in a consolidated Excel file by themes, double checked and grouped by the thematic areas of WHO. Specifically, each of the pandemic preparedness plans, as well as Covid‐19 specific policy and other measures were analysed as follows: first, assessment if the material was Covid‐19 related, second, content‐analysis of the documents on the health workforce, which was included if covering workforce capacity and/or skill‐mix. For the pandemic preparedness plans, all content related to Covid‐19 and the health workforce on workforce capacity expansion, skill‐mix and task‐shifting was extracted, as well as planning with reference to WHO's human resources in health scenario. WHO provides a checklist for the management for pandemic influenza (which includes aspects related to health service personnel). The checklists recommend recruiting retirees, nonpracticing healthcare professionals, volunteers, and to create plans for minimum staffing levels.^21^, ^22^ The pandemic preparedness plans were analysed separately as they were an indication of plans ahead of epidemics in contrast to the remaining gathered data that includes measures taken (policies, other) during the Covid‐19 pandemic. Third, the material identified during the search was grouped within the two thematic areas by subthemes (i) workforce capacity: expansion of work hours, recruitment of additional personnel within and outside the health sector, reassignment and reallocation of staff, and support measures (e.g., child care); (ii) skill‐mix and task‐shifting: existence of a policy on task‐shifting, training and/or education regarding Covid‐19. In a final step, the findings were compared by themes and subthemes across states and in relation to the federal level in order to identify the extent to which measures were similar or divergent across states and the extent to which federal policies and measures were taken up in the regions.

## 3 RESULTS

The analysis covered the websites of the Federal Ministry of Health and the public health authority (RKI), as well as the websites of the respective ministries and public health authorities of the 16 states. All states had pandemic preparedness plans and websites with information on Covid‐19 related policies and measures available.

### 3.1 Pandemic preparedness plans

In Germany, pandemic preparedness plans exist at the federal and state levels. The federal pandemic preparedness plan has been developed by the RKI, for any health‐related pandemic and was supplemented with a Covid‐19 specific section.^23^, ^24^, ^25^ Each state had a pre‐existing pandemic preparedness plan and most refer to the federal plan and focus on a possible influenza (H1N1) pandemic. The states varied in the extent to which the plans covered Covid‐19; seven included Covid‐19 (see Table 1).

**TABLE 1:** Pandemic preparedness plans in Germany at federal and state levels: existence, health workforce covered and skill‐mix

Federal/state level	Existence of pandemic preparedness plan ✓ Covid‐19 covered; (✓) Covid‐19 not covered	Health workforce mentioned	Health workforce scenario planning by WHO scenarios	Expand workforce surge capacity	Skill‐mix and task‐shifting ✓ skill‐mix covered (✓) skill‐mix not covered but education	Reference
Federal level	✓	✓		✓	✓	^23^, ^24^, ^25^
State level						
Baden‐Württemberg	**✓**	**✓**		**✓**	**✓**	^26^
Bavaria	**(✓)**	**✓**	**✓**	**✓**	**✓**	^27^
Berlin	**(✓)**	**✓**				^28^
Brandenburg	**✓**	**✓**		**✓**		^29^
Bremen	**(✓)**	**✓**		**✓**	**✓**	^30^
Hamburg	**(✓)**	**✓**		**✓**	**(✓)**	^31^
Hesse	**(✓)**	**✓**	**✓**	**✓**	**(✓)**	^32^
Lower Saxony	**(✓)**	**✓**		**✓**	**(✓)**	^33^
Mecklenburg‐Vorpommern	**✓**	**✓**		**✓**		^34^
North Rhine‐Westphalia	**(✓)**	**✓**		**✓**	**(✓)**	^35^
Rhineland‐Palatinate	**✓**	**✓**		**✓**	**✓**	^36^
Saarland	**✓**	**✓**	**✓**	**✓**	**✓**	^37^
Saxony	**✓**	**✓**		**✓**	**✓**	^38^
Saxony‐Anhalt	**✓**	**✓**		**✓**	**✓**	^39^
Schleswig‐Holstein	**(✓)**	**✓**		**✓**	**(✓)**	^40^
Thuringia	**(✓)**	**✓**	**✓**	**✓**	**✓**	^41^

The health workforce is mentioned in all 16 states and in the federal preparedness plans. Four state preparedness plans refer to health workforce planning according to the WHO scenarios described in checklists for pandemic influenza.^21^, ^22^ Lower Saxony was the only state with a staffing plan (overview of number of health workers but no minimum staffing levels).^33^ The federal and all state pandemic preparedness plans (except Berlin) have included measures to increase the workforce capacity. The plans offer several and diverse strategies to expand the health workforce, but some suggested actions have been mentioned by several states: recruitment of students/workers in training (6 states), cancelling of elective procedures (6/federal), cohort care (continuous care of infected/not infected patients by the same staff) (6/federal), reallocation of staff from other units (7/federal), recruitment of retirees (4), early discharge of patients (3), recruitment of volunteers or temporary staff (4), and use capacities of rehabilitation clinics (4). See Supporting Information Materials S1 for more detail.

Skill‐mix and task‐shifting was not mentioned directly in any of the states, but indirectly addressed by 13 plans and covered training of health workforce (12/federal) and some suggest reallocation of staff from other units or specialty areas (7/federal).

Some of the states have identified additional measures impacting indirectly on the health workforce. In Bremen only urgent or emergency cases were suggested to be admitted for inpatient care, and nursing home care shall be undertaken by relatives.^30^ Hesse's and Lower Saxony's plans suggest to have an on‐call duty activated that allows for mobile patient care at home by physicians.^32^, ^33^ In Mecklenburg‐Vorpommern, federal army hospitals were suggested to be used to increase capacities.^34^ Rhineland‐Palatinate's plan describes the recruitment of ‘external personnel’,^36^ and in Saarland medical teams by the Association of Statutory Health Insurance Physicians are planned to be on duty to support practicing health professionals.^37^

### 3.2 Policies and measures to expand workforce surge capacity

All 16 states have introduced measures to expand the health workforce, yet, they varied considerably (see Table 2).

**TABLE 2:** Strategies at federal and state level to expand workforce capacities

Federal/state level	Expansion of work hours of existing health professionals	Recruitment of retirees (R), medical/nursing students (MS, NS), foreign trained (FT) ‐physicians (P)/‐nurses (N)	Recruitment of volunteers, trained staff working outside health sector—nurses (N), physicians (P), health professionals (HP)	Reassignment and reallocation of staff	Support measures: child daycare (C), care of care recipients (CR)	Other
Federal level	**✓**					
State level						
Baden‐Württemberg			**✓** N		**✓** C	
Bavaria		**✓** R, MS, HP on parental leave	**✓** N, P, HP	**✓** within hospital; deployment of suitable physicians from hospitals/primary care to secure emergency care	**✓** C	**✓** civil servant candidates
Berlin			**✓** HP	**✓** within hospital	**✓** C	
Brandenburg		**✓** FT‐P (Poland)			**✓** C	**✓** military
Bremen			**✓** HP		**✓** C, CR	
Hamburg	**✓** Max. 12 h/day	**✓** MS	**✓** HP		**✓** C, CR	**✓** military, Red cross
Hesse		**✓** MS			**✓** C	
Lower Saxony	**✓** Max. 12 h/day, Max. 60 h/week			**✓** personnel from MDK to nursing homes	**✓** C, CR	
Mecklenburg‐Vorpommern	✓ Max. 12 h/day	✓ MS, FT‐P (Poland)		✓ university hospital staff for ‘fever outpatient clinics’; reallocation of staff within hospital cluster	✓ C	
North Rhine‐Westphalia		**✓** MS, FT‐P (Poland)			**✓** C	
Rhineland‐Palatinate	**✓** nursing homes, home care service	**✓** R, FT‐N (deployment as nursing assistance during recognition of qualification)	**✓** N, HP	**✓** within hospital; across nursing homes and home care services	**✓** C	
Saarland		**✓** R	**✓** N, P		**✓** C	
Saxony	**✓** Max. 12 h/day			**✓** within hospitals	**✓** C	
Saxony‐Anhalt					**✓** C	
Schleswig‐Holstein			**✓** N	**✓** within hospitals	**✓** C	
Thuringia					**✓** C	

Abbreviation: MDK, Medical Review Board of the Statutory Health Insurance Funds.

One strategy was to expand the working hours per day. In Germany, the working hours act (Arbeitszeitgesetz [ArbZG]) determines the maximum eligible working hours per day and week, including rest times. An amendment of the working hours act (ArbZG sect. 14 para. 4) by the Federal Ministry of Labour and Social Affairs and a decree (COVID‐19‐ArbZV) was enacted in five states. Four states extended the maximum working hours explicitly to 12 h/day.^42^, ^43^, ^44^, ^45^, ^46^ In Rhineland‐Palatinate, the focus was on expanding working hours for staff in nursing homes and mobile long‐term care services, a setting with staff shortages already prior to the pandemic.^47^, ^48^

Eight states implemented measures beyond the practicing health professionals. This included attracting retirees (e.g., medical doctors, nurses) back to the workforce, recruiting medical students and providing licenses for foreign‐trained health care professionals. Three states initiated measures to recruit retirees back to the workforce.^47^, ^49^, ^50^, ^51^, ^52^ In five regions medical students were identified as a resource to support the health workforce and to be recruited.^50^, ^53^, ^54^, ^55^ Three states provided temporary licenses for physicians trained in Poland to solve a problem of recognition of Polish degrees that existed at the same time.^56^, ^57^, ^58^, ^59^ Active recruitment of Polish physicians for the purpose of Covid‐19 was not described. Rhineland‐Palatinate authorised work permissions for foreign trained nurses waiting for recognition of their diplomas to work as nursing assistants.^60^ Bavaria identified multiple measures to expand workforce capacity and was the only state that considered recruiting health professionals on parental leave.^49^, ^50^, ^61^, ^62^, ^63^, ^64^ Medical students in Bavaria were recruited by multiple channels on state and federal level, for instance by a joint call of the Ministry of Health and the Bavarian Medical Association but also by a nationwide call of the RKI.^65^

Eight states started measures to recruit nonpracticing healthcare workers. Of those, five states explicitly recruited nurses, two focused on physicians and five advertised for health professions in general.^49^, ^61^, ^62^, ^63^, ^66^, ^67^ At federal level no initiative was identified to recruit nurses but the online platform ‘Pflegereserve’ (care reserve) was mentioned as this platform was used in several states to recruit nurses. Rhineland‐Palatinate and Schleswig‐Holstein are examples of the few states in Germany with a nursing chamber and a register of nurses, and offered nurses working outside the health care sector to register.^68^, ^69^

Seven states used the strategy reassignment and/or reallocation of staff to increase health workforce capacities. Most often the reallocation was applied within hospitals in combination with cancelling elective procedures which freed up staff for the care and treatment of patients with Covid‐19.^50^, ^51^, ^68^, ^70^, ^71^, ^72^ In Lower Saxony staff from the Medical Review Board of the Statutory Health Insurance Funds (MDK) supported nursing homes as auditing tasks of the MDK were stopped during this time.^70^ In Rhineland‐Palatinate an exchange of staff across nursing homes and home care service was described to secure the care of care recipients in both settings.^48^ In Mecklenburg‐Vorpommern ‘fever outpatient clinics’ were implemented in order to protect and release pressure from the ambulatory care setting, which was in Germany mostly the first point of contact for patients with symptoms.^73^ In Mecklenburg‐Vorpommern regional hospital clusters were established and the opportunity to reallocate staff within each cluster was mentioned.^74^ At federal level, the reallocation of staff was supported implicitly as hospitals received financial compensation of 560€/day for unoccupied beds (the reference year 2019), so that staff can focus more on patients with Covid‐19. This measure was determined with an amendment of the Hospital Financing Act (Krankenhausfinanzierungsgesetz), time‐bound until September 2020.^75^ Two other measures are time‐bound (determined in the respective decrees): expansion of working hours (period of few months) and licenses for foreign trained staff (up to spring 2021).

In all 16 states, additional support measures were implemented, mainly the provision of day care for children of health professionals (identified as ‘system‐relevant professions’, in addition to children from firefighters, school teachers, supermarket personnel and others).^76^ Even though the measure was implemented in all states, each state had its own decrees and there was no federal regulation. In addition, three states offered day care for health professionals with dependent, frail family members.^77^, ^78^, ^79^

Three states made use of additional support measures to expand workforce capacities. German armed forces provided support in nursing homes (Hamburg) and with contact tracing (Brandenburg). In Bavaria, civil servant candidates also worked in contact tracing teams.^52^, ^80^, ^81^ The German Red Cross supported nursing homes in Hamburg with testing residents and staff.^82^

### 3.3 Policies on task‐shifting

At federal level, the infection control law (§ 5a) authorises task‐shifting in case of nationwide epidemics^83^ (Table 3). According to the law, task‐shifting of medical tasks is authorised to nurses and emergency paramedics independently under the following conditions: the competencies must be available to carry out tasks independently, adequate documentation is required, the professional must inform the responsible or treating physician and the care is performed for patients with no severe conditions. There was no additional information what specific tasks the law covers, how the competencies should be assessed and by whom and how task‐shifting should be implemented in practice.

**TABLE 3:** Policy and training related to skill‐mix and task‐shifting during Covid‐19

Federal/state level	Policy on task‐shifting	Further details	Training/education related to task shifting/skill‐mix	Further details
Federal level	**✓** (§5a Infection control law)	Task‐shifting of medical tasks to nurses and paramedics if (i) sufficient competencies, (ii) adequate documentation, (iii) physician informed, (iv) for patients with no severe condition		
State level				
Baden‐Württemberg				
Bavaria				
Berlin			**(✓)** (nursing homes)	
Brandenburg			**(✓)** (nursing homes)	
Bremen				
Hamburg				
Hesse			**(✓)** (hospitals)	
Lower Saxony				
Mecklenburg‐Vorpommern			**(✓)** (pharmacies)	
North Rhine‐Westphalia			**✓** (medical students)	Medical students: possibility to start working prior to medical exam, supervisory structures required
Rhineland‐Palatinate	**✓** (task‐shifting in long‐term care)	Task‐shifting of ‘medical nursing care’ and basic nursing care from nurses to lower‐qualified, suitable staff at discretion of nursing management	✓ (workforce expansion in intensive care)	ICU training for nurses and doctors in intensive care
Saarland				
Saxony			**✓** (medical students)**(✓)** (nursing homes)	Medical students: possibility to start working prior to medical exam; Nursing homes: training of staff, including online training
Saxony‐Anhalt	**✓** (further on §5a on emergency paramedics)	Additional details of how and under what circumstances medical tasks can be carried out by paramedics independently	**✓** (education, emergency paramedics)	Competencies for task‐shifting as per basic education and operating procedures
Schleswig‐Holstein			**(✓)** (training of staff)	Training, for example, in hygiene; use of infected staff in case of shortage and after additional training
Thuringia				

Abbreviations: ICU, intensive care unit; ✓, yes, related to Covid‐19 and skill‐mix/task‐shifting; (✓), partly yes, related to Covid‐19 but unclear if skill‐mix/task‐shifting.

At state level, only Saxony‐Anhalt further specified the infection control law (§ 5a) and adopted a joint decree that provided additional information supplementing the federal law for emergency paramedics. It states that emergency paramedics possess the competencies (as per emergency paramedics law) and as per treatment pathways and standard operating procedures to perform certain medical tasks independently during pandemics. It is highlighted that each paramedic should only perform tasks and skills as per personal abilities and skills acquired, which depends on the work experience and additional training. These skills are to be determined a priori by the medical director of the rescue service. If task‐shifting has occurred in practice, adequate and immediate communication and documentation to a medical doctor is required, for example, the director of the rescue service and/or responsible physician in a hospital to which the patient is transported.^84^

No further information was identified on task‐shifting from physicians to nurses (as covered in the federal law), neither in Saxony‐Anhalt, nor in any of the other states.

All provisions under § 5 of the infection control law, including task‐shifting, are in place in light of Covid‐19 until 31 March 2021 and will be evaluated thereafter.^85^

One state, Rhineland‐Palatinate, has made provisions to specify task‐shifting in ambulatory long‐term care from nurses to lower qualified staff, for example, nursing assistants (time‐bound until 31 March 2021). Specific nursing tasks which are usually reserved to the nursing profession as well as basic nursing care can be delegated to lower‐qualified staff at the discretion of the management if the competencies and expertise are available.

Table 3 shows training and education related to skill‐mix and task‐shifting. At the federal level, no information was provided what the training and education requirements for nurses and paramedical staff should be regarding the law on task‐shifting. In Saxony‐Anhalt, the law on task shifting from physicians to emergency paramedics (§ 5) also referred to the training and competencies, which are to be covered by the standard basic education and standard operating procedures.^84^

In Rhineland‐Palatine, training opportunities and financing directly related to upskilling within a profession were provided via information for nurses and physicians in intensive care, to expand capacity in intensive care units (ICUs). Training is offered by 24 institutions and financed by the state. There is a 180 h ‘rapid’ training in intensive care nursing, with the overall aim to expand capacity for an additional 2000 beds.^69^, ^86^ North Rhine‐Westphalia focused on training of medical students to expand workforce capacity.^87^

Several states introduced provisions for the training or further education of health professionals, either setting‐specific, profession‐specific or for all health professionals. It was not clear, however, if the training was also partly related to skill‐mix, and therefore are marked as such in Table 3. Examples included training for staff working in nursing homes. In Brandenburg for instance, training was operated via telephone hotlines by the MDK on Covid‐19‐related content, such as isolation, hygiene, handling of suspected cases, among others.^88^ In Mecklenburg‐Vorpommern, training focused on pharmacies and hygiene, isolation and suspected cases.^89^, ^90^ In Schleswig Holstein, training covered all staff, whereas in Rhineland‐Palatinate, suggestions focused on the training of nurses: training (modules) were established by the regional nursing chamber and coordinated (in five educational institutions) plus online resources.

## 4 DISCUSSION

As a response to the Covid‐19 pandemic, Germany used various health workforce strategies to strengthen and expand the workforce. This analysis shows that strategies varied largely across the 16 states. Most action was applied on expanding health workforce capacities but limited on skill‐mix and task‐shifting. Pandemic preparedness plans exist in all 16 states and varied considerably. All covered health workforce to at least some extent, but most were not very specific and the majority were not updated in light of the Covid‐19 situation.

Since 2005, all 16 states developed their own pandemic preparedness plans including state‐specific adjustments.^91^ Only seven states made specific reference to Covid‐19 in the plans. No information was found why some states updated their plans and others not and to which extent the plans were followed in the states' responses to the pandemic and lessons learned. From experience with the influenza H1N1 in 2009, the RKI concluded that Germany's ‘preparedness was largely in an advanced stage and most probably contributed to successful control’ of the 2009 pandemic, however, communication and logistics of vaccines were topics most in need of improvement (ibid). An evaluation of how well Germany and each state were prepared during the Covid‐19 pandemic, if pandemic preparedness plans were implemented, and which measures have been effective in practice, should be considered in future revaluations and for further research. An external evaluation of countries' preparedness to respond to health emergencies is provided by the WHO's Joint External Evaluation (JEE), a tool recommended to all WHO's member states to evaluate and monitor international health regulations capacity.^92^ Two‐thirds of all member states completed or planned the JEE in 2019, and results are available for four EU countries.^93^, ^94^ Germany has also conducted the JEE in 2019 but the report has not published to date.^95^

There were marked differences between the states in the extent to which health professionals were covered in the preparedness plans and workforce planning. It is unclear why there were these differences. It could be hypothesized that states governed by political parties with a stronger role for planning resulted in preparedness plans being more developed than in states governed by parties that emphasize a less intense role for the state. One example is Bavaria which has planned and implemented a range of workforce measures and is traditionally governed by a conservative party. Other reasons may be differences in the public health tradition in the states and expertise in workforce planning among the teams writing pandemic preparedness plans. There needs to be more research into the underlying reasons for these differences across states.

An analysis of 43 European countries and Canada showed a variety of strategies to increase health workforce capacity. For Germany five out of eight measures were identified and these results are in line with our study. However, the study also showed that there were measures in place in other countries that were not applied in Germany, for instance, recruitment of inactive health professionals and use of armed forces.^10^ In contrast, our analysis shows that both strategies indeed were used in a few states, first, nonpracticing health professionals were called to register at online platforms, second, armed forces supported the states Brandenburg and Hamburg. One reason for the differences may be that the international study focused on measures at the federal level and those implemented in many states, whereas our analysis provided a state‐by‐state analyses and was conducted somewhat later, and more measures were in place at the time of search.

Our analysis is largely in line with the findings from the international OECD study, which showed Germany mobilised healthcare students and foreign trained health workers.^11^ Both measures were not nationwide strategies but applied in five (medical students) and three states (foreign health professionals), respectively. In the OECD study, Germany was not listed regarding the following three measures: recruitment of retirees and nonpracticing health workers, reallocation of staff to places with greater need, existing or new reserve list. The first two aspects were, however, identified during our search. An official reserve list with available health professionals does not exist in Germany. However, there were several options to register voluntarily, mainly for nonpracticing health professionals and medical students. One example is the website of ‘Pflegereserve’ which was originated by stakeholders such as ‘Deutscher Pflegerat’ (German nursing council). Nine states officially refer to this website. Nurses, nursing associates and health care assistants can register but also hospitals and nursing homes which can contact those registered in case of need.

Reallocation of staff within hospitals was a strategy mentioned in five states. This was often combined with the cancellation of elective procedures which was reported by 72.4% of healthcare professionals surveyed (*n* = 2.827) in Germany in spring 2020.^96^ Hence, freed up staff could take care of Covid‐19 patients. The shortage of staff on ICUs is critical because reallocation may be more difficult as patients and tasks on ICU are more complex and take‐over of tasks by nurses and physicians without training in intensive care is not fully applicable. In Germany, a survey from 2017 among 446 physicians revealed that 22% of ICU floors had to close beds and the main reason was a lack of trained ICU nurses.^97^

The Covid‐19 incidence levels per 100,000 inhabitants varied greatly among regions and districts in Germany which led to large differences in the number of Covid‐19 patients in hospitals.^98^ Reallocation of staff across the country from less affected to more affected areas could be an opportunity, but no information was identified that this has taken place. In Mecklenburg‐Vorpommern it was mentioned that clusters of hospitals were established and a cluster manager coordinates the ventilators and the required personnel.^74^

Limited action on skill‐mix and task‐shifting resembles the situation before the pandemic. A study by Maier and Aiken^99^ showed that task‐shifting between the medical and nursing profession was limited in Germany in comparison with 38 other countries. The federal law on infectious control measures stipulates the possibility to implement task‐shifting from physicians to emergency paramedics and nurses during Covid‐19. However, no state further specified this law on nurses. Only one state, Saxony‐Anhalt, referred to this provision regarding emergency paramedics. It is not known if and to which extent task‐shifting has occurred during the Covid‐19 pandemic and how it has been implemented in practice. Task‐shifting has shown to occur informally in daily practice if there are unmet need or care requirements, as shown in the Netherlands or Spain (ibid). It would be relevant for Germany to analyse if this has been the case during the pandemic and how to strengthen the professions and collaborative care in this regard.

Differences across states exist, as shown by the pandemic preparedness plans, expansion of workforce capacities, and skill‐mix and task‐shifting. This represents the federal structure of Germany. One example how differently measures were implemented is the deployment of medical students—the location of work differed (hospitals or public health institutes) and the tasks (support of patient care, contact tracing, perform Covid‐19 tests).

It is unclear why the measures differ considerably among the states, and whether the different approaches per state have an overall impact on Germany's Covid‐19 response. Germany ranked only 22nd in terms of the category health system response (including capacity in hospitals and personnel deployment) in the GHS index before the Covid‐19 pandemic.^16^ The authors of the GHS index recommend that every country should have an updated ‘health workforce strategy’ (ibid). However, case studies for Germany show a lack of an integrative health workforce governance between federal and local levels.^100^ Another reason for different measures across states could be coordination problems. This was described by Greer et al.^101^ for several federal countries, also for Germany regarding workforce capacity. The authors describe potential solutions such as informal communication and coordination between decision maker and transparency regarding decisions (ibid). In Germany, the federal government, together with the 16 state representatives have discussed periodically Covid‐19 measures, but implementation is the responsibility of the states and can be modified.

A limitation of our study is that not all relevant information may have been identified, for instance if not provided on the websites studied or not findable. Furthermore, only a limited statement can be made on the extent to which the measures have been implemented in practice. The search was conducted in May 2020 during the end of the first wave of the pandemic, hence, can only provide information for this time period. The second wave to date has shown more infections and patients being hospitalised and state measures may have been different and are still partly ongoing, which are not covered in this study. Yet, the study is the first of its kind which has systematically analysed state‐level responses on Covid‐19 from a health workforce perspective.

## 5 CONCLUSION

This research demonstrated the importance to analyse not only federal but also in detail state level measures in countries. Germany's states have implemented a range of different strategies to expand their health workforce during the first wave of Covid‐19, covering extra working hours, retirees and student recruitment, among others. Many pandemic preparedness plans were not Covid‐19 specific and planning for health professional capacity expansion was limited, as were strategies on skill‐mix and task‐shifting. Overall, there was little evidence of harmonisation, evaluation and limited planning to expand health workforce capacity. These results are of relevance for federal countries with a decentralised workforce governance structure. Lessons from Covid‐19 first wave should feed into subsequent waves and future pandemics.

## CONFLICT OF INTEREST

The authors declare that there is no conflict of interest.

## ETHICS STATEMENT

No ethical approval was obtained because the data used were freely available and no personal data were used.

## Supporting information

Supplementary Material

## Data Availability

Data sharing not applicable to this article as no datasets were generated or analysed during the current study.
