# Supplementary material for: Health workforce response to Covid‐19: What pandemic preparedness planning and action at the federal and state levels in Germany? Germany's health workforce responses to Covid‐19
Source: Int J Health Plann Manage. 2021 Mar 18;36(Suppl 1):71–91. doi: 10.1002/hpm.3146 (PMC8250947; doi:10.1002/hpm.3146)
Supplement: Supplementary file 1 — Supplementary Material [file HPM-36-71-s001.docx]

**Supplement 1:** Pandemic preparedness plans in Germany at national and state levels: existence, health workforce covered, and details on skill-mix and task-shifting

| Federal/State level | Existence of pandemic preparedness plan  ✓ Covid-19 covered;  (✓) Covid-19 not covered | Health workforce mentioned | Health workforce scenario planning by WHO scenarios | Expand workforce surge capacity | Skill-mix and task shifting | Reference |
| --- | --- | --- | --- | --- | --- | --- |
| Federal level | **✓** | **✓** |  | Guidelines for workforce planning, cohort care (continuous care of infected or not infected patients by the same staff), recruitment of medical students or workers that are still in training, cancellation of elective procedures | (ongoing) education of health workforce, shifting and special education of health personnel from other health departments (that is free due to cancellation of elective procedures) | 23-25 |
| State level |  |  |  |  |  |  |
| Baden-Württemberg | **✓** | **✓** |  | recruitment of medical students, of medical staff still in training or of medical staff not working in the health sector, physical rehab institutions can take over inpatient care, cancelling of elective procedures, early dismission of patients, | Shifting and special education of health workforce from other health departments (e.g. dermatology, ophthalmology), ongoing training of health care personnel | 92 |
| Bavaria | **(✓)** | **✓** | **✓** | List of licensed retirees, recruitment of medical students, health workers in training and retirees, cancelling of elective procedures, early discharge of patients | Training of health workforce, shifting and special education of health workforce from other health departments | 93 |
| Berlin | **(✓)** | **✓** |  |  |  | 94 |
| Brandenburg | **✓** | **✓** |  | Recruitment of temporary and voluntary staff via medical schools, employment office or the state’s medical association, cohort care |  | 95 |
| Bremen | **(✓)** | **✓** |  | Inpatient care only for urgent cases, ambulatory care preferably through relatives instead of health care personnel, cancelling of elective procedures, early dismission of patients, cohort care | Training of health workforce, clinic workforce to be reallocated to internist and pediatric wards | 27 |
| Hamburg | **(✓)** | **✓** |  |  | Specific training of occupational health workers, training for health workers | 96 |
| Hesse | **(✓)** | **✓** | **✓** | Ambulatory 24h on-call emergency duty, cancelling/moving of elective procedures, cohort care | Training of the health workforce | 28 |
| Lower Saxony | **(✓)** | **✓** |  | Staffing plan, plans for on-call duty, care facilities shall stay open as long as possible to minimize overload in hospitals, recruitment of voluntary staff, suspension from duty in case of sickness to protect the healthy personnel | Training of health workforce | 26 |
| Mecklenburg-Vorpommern | **✓** | **✓** |  | Recruitment of temporary and voluntary staff via medical schools, employment office or the state’s medical association, cohort care, physical rehab and prevention institutions can take over inpatient care as well as inpatient wards of the federal army (Bundeswehr), suspension from duty in case of sickness to protect the healthy personnel |  | 29 |
| North Rhine-Westphalia | **✓** | **✓** |  | Inpatient care only for urgent cases | Training of health workforce | 97 |
| Rhineland-Palatinate | **✓** | **✓** |  | Recruit “external personnel” | Shifting and special education of health personnel from other health departments | 30 |
| Saarland | **✓** | **✓** | **✓** | Recruitment of “reserve personnel” (medical students, staff in training, retirees), recruit volunteers; moving elective procedures, physical rehab institutions can take over inpatient care, | Workshops for health professionals, substitution of practitioners by on-duty medical units through the Association of Statutory Health Insurance Physicians | 31 |
| Saxony | **✓** | **✓** |  | Recruitment of medical students or staff still in training, cancelling of elective operations, cohort care, | Training of health workforce, shifting and special education of health personnel from other health departments | 98 |
| Saxony-Anhalt | **✓** | **✓** |  | List of retired physicians and re-recruitment, recruitment of medical students and workforce still in training, cancelling of elective surgery, physical rehab institutions take over inpatient care, early dismission of patients | Ongoing workshops and training for healthcare personnel, especially for personnel that will be shifted from other units | 99 |
| Schleswig-Holstein | **(✓)** | **✓** |  | Recruitment of physicians in administrator positions (only for vaccination strategy), Cohort care, | Training of health workforce | 100 |
| Thuringia | **(✓)** | **✓** | **✓** | Expansion of working hours, measures for workforce recruitments and shortage management, recruit workforce still in training and retirees, cancelling of elective procedures, suspension from duty in case of sickness to protect the healthy personnel | Workshops and training for of health workforce, shift personnel from other departments | 101 |
